# A Numerical Investigation into the Effect of Homogeneity on the Time-Dependent Behavior of Brittle Rock

**DOI:** 10.3390/ma14226818

**Published:** 2021-11-11

**Authors:** Hao-Zhe Chen, Zhu-Shan Shao, Dong-Dong Jin, Zhe Zhang, Dong-Bo Zhou

**Affiliations:** 1 School of Civil Engineering, Xi’an University of Architecture & Technology, Xi’an 710055, China; chenhaozhe515@foxmail.com (H.-Z.C.); zzhe0315@xauat.edu.cn (Z.Z.); zdb@xauat.edu.cn (D.-B.Z.); 2 Shaanxi Key Lab of Geotechnical and Underground Space Engineering, Xi’an University of Architecture & Technology, Xi’an 710055, China; jindongdong@xauat.edu.cn; 3 School of Science, Xi’an University of Architecture & Technology, Xi’an 710055, China

**Keywords:** creep, homogeneity, stress level, steady creep rate, dilatancy, failure pattern

## Abstract

To investigate the brittle creep failure process of rock material, the time-dependent properties of brittle rocks under the impact of homogeneity are analyzed by the numerical simulation method, RFPA-Creep (2D). Deformation is more palpable for more homogeneous rock material under the uniaxial creep loading condition. At a low stress level, diffusion creep may occur and transition to dislocation creep with increasing applied stress. The law for increasing creep strain with the homogeneity index under a constant confined condition is similar to the uniaxial case, and dislocation creep tends to happen with increasing confining pressure for the same homogeneity index. The dilatancy index reaches its maximum at a high stress level when rock approaches failure, and the evolution of the dilatancy index with the homogeneity index under the same confining pressure is similar to the uniaxial case and is more marked than that under the unconfined condition. Both uniaxial and triaxial creep failure originate from the ductile damage accumulation inside rock. The dominant shear-type failure is exhibited by uniaxial creep and the conventional compression case presents the splitting-based failure mode. Under confining pressure, the creep failure pattern is prone to shear, which is more notable for the rock with higher homogeneity.

## 1. Introduction

Excavations of rock mass at great depth for mining, tunneling, etc. could be accompanied by server dynamic disasters such as rockbursts, which commonly take place in a sudden manner during excavations [1,2,3]. In most cases, the rheology of surrounding rock mass manifests as obvious creep for the delayed rockburst under the action of a strong time effect [4], especially when a deep rock mass is subjected to high in situ stresses, and the delayed duration of the rockburst events ranges from several hours to days, even months [5,6,7]. With the evolution of time-dependency, the creep failure of hard and brittle surrounding rock easily triggers the time-delayed rockburst, threatening the safety and stability during construction of deep rock engineering.

It is known that creep of brittle rocks can be defined as the time-dependent irreversible deformation which occurs during the action of a constant applied stress lower than short-term strength [8,9]. Currently, the various research approaches for time-dependent behavior of brittle rocks are mainly divided into three aspects: theory, experiment and numerical simulation [10,11,12,13,14]. Brantut et al., 2012 and Li et al., 2019 explored the relationship between brittle creep behavior and crack growth on a microscopic scale by extending the same micromechanical theoretical model [15,16,17]. Shi et al., 2018 analyzed the damage evolution inside a cuboid-shaped sandstone specimen reflected by the real-time recorded spacial development of AE activities during the creep loading test [18]. Tang, 2013 obtained the surface creep deformation of rock specimen based on strain fields that are measured using DIC (digital image correlation) technology [19]. Although the initiation of the numerical method in measuring the mechanical behavior of rock material is relatively recent, it has been widely adopted and constantly developed by numerous scholars and engineers including the finite difference methods (FDM), finite element methods (FEM), boundary element methods (BEM) and meshless methods [20,21,22,23,24,25], etc., and a few numerical studies which have focused on the creep property of rock. Lockner and Madden, 1991b managed to depict the characteristics of accelerating creep phase and predict the proper stress sensitivity of creep rate by the developed numerical multiple-crack interaction model [26]. According to the precious progressive damage model [27], Amitrano and Helmstetter, 2006 further established the time-independent model and gained the spacial distribution of damage of rock at different stress levels by numerical analysis [8]. As a valid numerical analysis tool, RFPA (realistic failure process analysis) comprehensively considers the nonlinearity of the failure process and the heterogeneity of rock material, and it introduces the multiple critical factors such as temperature, moisture, etc., and a few applications on the study of rock time-dependency have been achieved [28,29,30,31]. Li et al., 2008 observed the creep failure process of rock during unconfined loading by introducing the constitutive model for microscopic elements under the time effect [32].

Therefore, in order to investigate the mechanism of brittle creep failure of rock material, with the aid of the numerical simulation method, RFPA-Creep (2D), the effect of homogeneity (the homogeneity indexes m of 1.5, 2, 2.5, 3 and 5) on time-dependent properties of brittle rock under uniaxial and triaxial creep loading is analyzed in this paper. The relationship between stress level and steady creep rate, the characteristics of dilatancy, the damage evolution and failure pattern of rock are discussed.

## 2. Model Descriptions and Setup

### 2.1. Brief Description of RFPA 2D (Creep)

**Figure 1:**
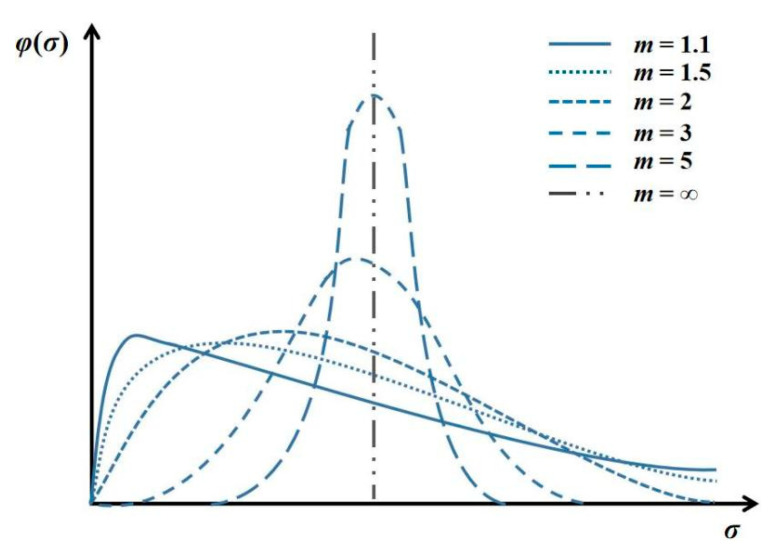
Mechanical property distribution for five various homogeneous specimens (both strength and elastic modulus follow the same distribution).

The two-dimensional finite mode, RFPA 2D, is a numerical simulation tool introducing the homogeneity index, which can be used to analyze progressive internal damage evolution until the macroscopic failure of brittle rock material, and the time effect is further considered by RFPA-Creep. From a microscopic angle, it is assumed that the model medium is composed of rectangular elements on the same scale and that the statistical distribution of elemental mechanical properties obeys a Weibull distribution [33]:

(1) $$\varphi \left(\alpha \right)=\frac{m}{\alpha_{0}}{\left(\frac{\alpha }{\alpha_{0}}\right)}^{m-1}exp-{\left(\frac{\alpha }{\alpha_{0}}\right)}^{m}$$

where *α* is a mechanical property where strength and elastic modulus are set as the same distribution; *α*_0_ is a mean value of the corresponding parameter and *m* refers to the homogeneity index of rock. The rock material becomes more homogeneous with a larger *m* (Figure 1) [28].

The mechanical behavior of microscopic elements is modeled by the approach on damage mechanics. The initiation of micro-scale failure occurs after the stress state of an element meets a strength criterion such as the Coulomb criterion. The elastic modulus of an element is assumed to degrade gradually during damage evolution and can be described by:

(2) $$E=\left(1-D\right)E_{0}$$

where *D* is the damage variable; *E* and *E*_0_ are the elastic modulus of the degraded and original element, respectively.

**Figure 2:**
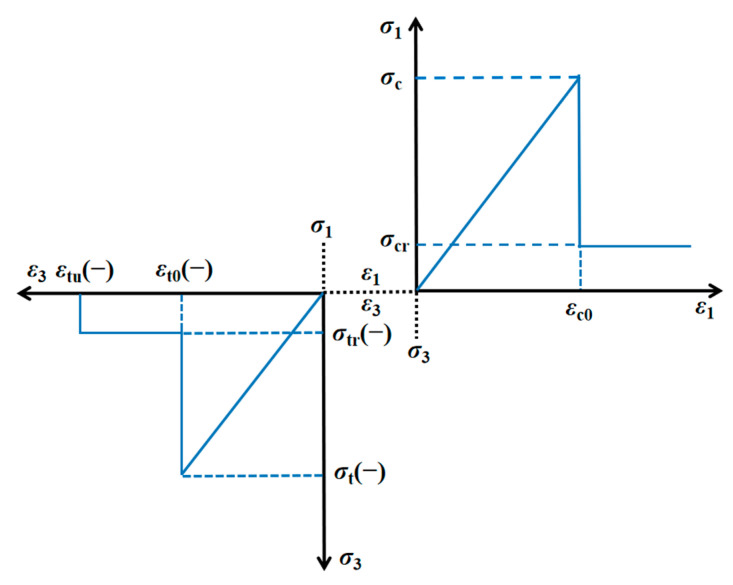
Elastic–brittle damage constitutive law of the micro element.

In this model, the compressive stress is set as the positive and the tensile stress is negative (Figure 2). Both shear and tensile failure patterns are considered where the former mode appears when the compressive stress of an element satisfies the Mohr–Coulomb failure criterion:

(3) $$\sigma =\sigma_{1}-\sigma_{3}\frac{1+sin\varphi }{1-sin\varphi }\geq \sigma_{c}$$

where *σ*_1_ and *σ*_3_ represent the major and minor principal stress, respectively; *σ*_c_ is the compressive strength and *φ* is the internal friction angle. An element fails in tensile pattern when the minor principal stress reaches and exceeds the tensile strength *σ*_t_, which is expressed as follows:

(4) $$\sigma_{3}\leq -\sigma_{t}$$

The damage variable of an element with a compressive and tensile state can be, respectively, described as [29]:

(5) $$D=\left\{ \begin{array}{l} 0\\ 1-\frac{\sigma_{\mathrm{cr}}}{E_{0}\varepsilon } \end{array}\begin{array}{c} \left(\varepsilon <\varepsilon_{c_{0}}\right)\\ \left(\varepsilon_{c_{0}}\leq \varepsilon_{r}\right) \end{array}\right.$$

(6) $$D=\{\begin{array}{c} 0\\ 1-\frac{\sigma_{\mathrm{tr}}}{E_{0}\varepsilon }\\ 1 \end{array}\begin{array}{c} \left(\varepsilon <\varepsilon_{t_{0}}\right)\\ \left(\varepsilon_{t_{u}}<\varepsilon \leq \varepsilon_{t_{0}}\right)\\ \left(\varepsilon \leq \varepsilon_{t_{u}}\right) \end{array}$$

where *σ*_cr_ and *σ*_tr_ are the residual compressive and tensile strengths, respectively; *ε*_c0_ and *ε*_t0_ are the threshold compressive and tensile strains, respectively; *ε*_tu_ is the ultimate tensile strain and *ε*_r_ represents the residual strain.

It should be noted that the tensile criterion is given priority over the Mohr–Coulomb failure criterion for elements in this model, owing to the short compressive strength generally far outweighing the tensile one for brittle rock material. The Mohr–Coulomb criterion is adopted when the tensile criterion is not met for an element.

**Figure 3:**
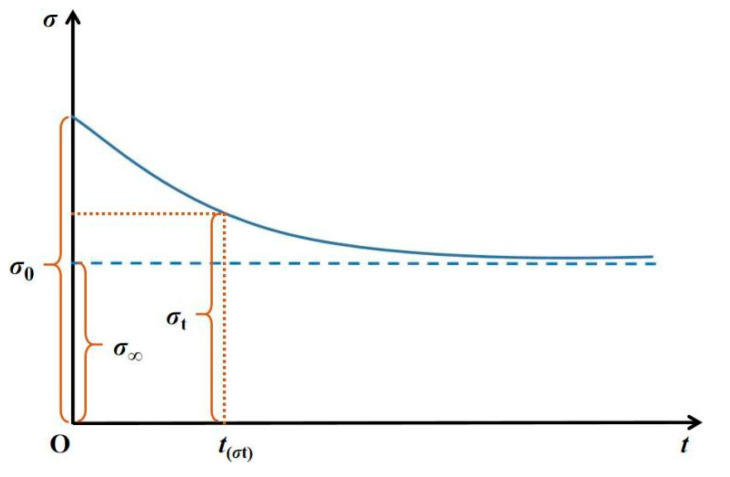
Constitutive law for strength degradation of the micro element.

In RFPA-Creep, the long-term strength of rock is introduced, representing that as time goes by, the strength of rock material degrades under a constant stress level. The creep constitutive relationship for microscopic elements is described as follows (Figure 3) [32]:

(7) $$\sigma =\sigma_{\infty }+\left(\sigma_{0}-\sigma_{\infty }\right)\exp\left(-Bt\right)$$

where *σ*_0_ and *σ*_∞_ separately represent the short-term and long-term strength of an element and *B* is the related attenuation coefficient.

It has been found that the degradation mechanism of elastic modulus is similar to that of strength [34]. Thus, it is assumed that the elastic modulus of rock obeys the same law:

(8) $$E=E_{\infty }+\left(E_{0}-E_{\infty }\right)\exp\left(-B^{\prime }t\right)$$

where *E*_0_ and *E*_∞_ represent the short-term and long-term elastic modulus of an element, respectively, and *B′* is the related attenuation coefficient. Both *B* and *B′* are assumed to be the same in this model.

**Figure 4:**
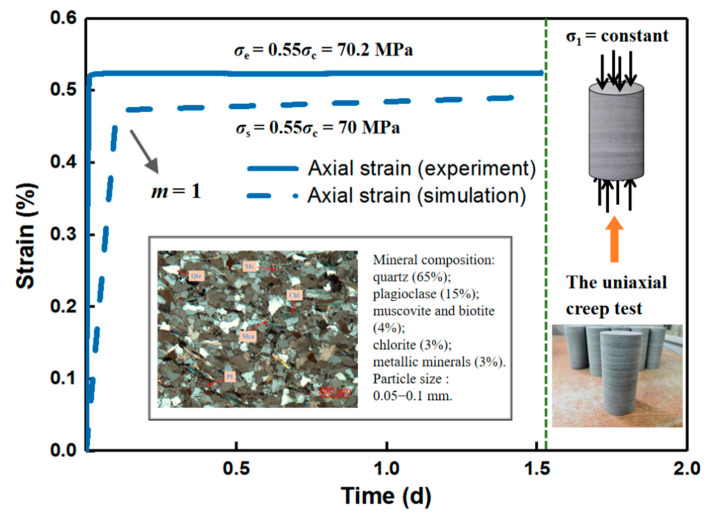
Comparison between experimental and numerical results.

Figure 4 exhibits the result of a uniaxial creep test for deep quartz sandstone taken from the Laobishan Tunnel at a low stress level set as 70.2 MPa and the corresponding numerical simulation. The specimen did not rupture and the evolution curve of axial strain with time nearly matched the numerical result. It can be observed that the homogeneity index *m* is 1, indicating that the rock specimen model is relatively heterogeneous. The significant difference between quartz sandstone specimens in mineral firmness and distribution and the particle size also gives rise to the heterogeneity of rock material on a microscopic scale. In addition, the mean value is used for the mechanical property of the micro element, and the size and shape, such as rectangle or triangle, of the element in model is invariable and regular, which is in contrast to the rock entity material whose micro-particle form and internal structure are uncertain. This may explain the slight difference in deformation between experimental and numerical results. Hence, RFPA-Creep (2D) can be applied to the simulation of time-dependent behavior concerning the homogeneity of rock material.

### 2.2. Specimen model setup

**Figure 5:**
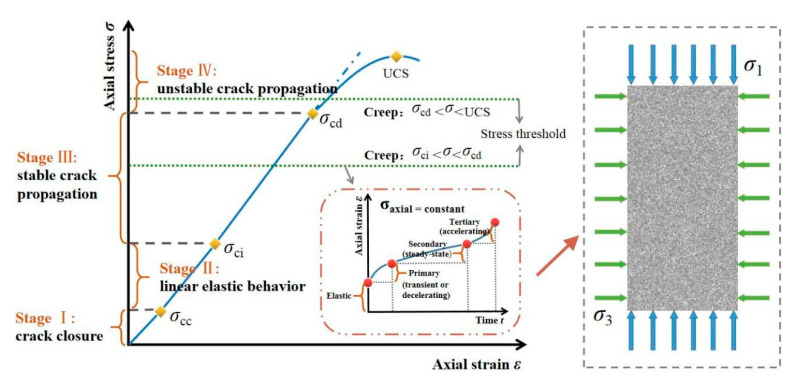
Stress–strain response and stages of rock during progressive fracture and classical creep behavior corresponding to the numerical model.

**Figure 6:**
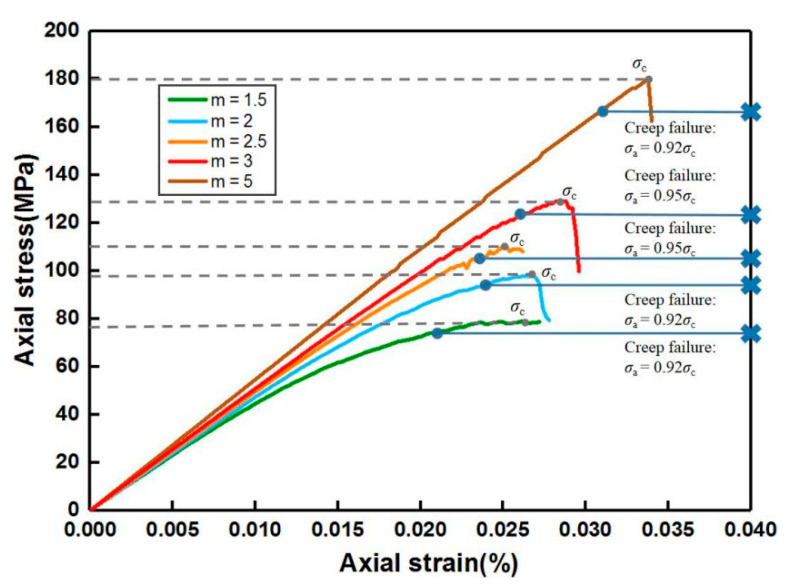
Uniaxial compressive simulation results for various homogeneity indexes.

In this study (Table 1 and Figure 5), the creep model dimension was 120 mm × 50 mm and the mesh was discretized into 240 × 100 = 24,000 elements. The specimen geometry was 100 mm × 50 mm (200 × 100 = 20,000 elements), and the loading plates with extremely high stiffness and homogeneity were set on the top and bottom of the specimen during the uniaxial creep loading, respectively. The plane stress compression was performed on all specimen models. The homogeneity indexes *m* of 1.5, 2, 2.5, 3 and 5 were selected for unconfined simulation conditions, and for triaxial cases with *m* = 2, 5, the confining pressure *P*_c_ (*σ*_3_) was set as 1.5 and 5 MPa. Based on the simulation results of the uniaxial compression process (Figure 6), the applied stress levels were set as 0.55*σ*_c_, 0.65*σ*_c_, 0.75*σ*_c_ and 0.85*σ*_c_ before failure at the last constant loading stage, which was also used for the triaxial creep simulation.

Furthermore, in order to calibrate the numerical specimen model, the partially input parameters of critical mechanical properties were set the same as those in the laboratory test or the setup in the model of RFPA 2D [28,32,35].

## 3. Numerical Results

During the uniaxial loading phase, the creep failure emerged when the last stress level reached 0.92*σ*_c_ for *m* = 1.5 (74 MPa), 2 (90 MPa) and 5 (166 MPa), and the applied loads when m was both both 2.5 (105 MPa) and 3 (124 MPa) were 0.95*σ*_c_. In the triaxial creep condition, when the confining pressure was 1.5 MPa, the specimen entered into the fracturing stage at applied stresses that were 96 MPa and 178 MPa (both *σ*_a_ = 0.91*σ*_c_) for *m* = 2 and 5, respectively, and when *P*_c_ = 5 MPa, the last load levels were 109 MPa (*m* = 2) and 206 MPa (*m* = 5), and both exceeded 0.9*σ*_c_. In addition, the duration for all specimens during the unchanging high stress level which led to the failure was within 2 d, except for the unconfined condition, with *m* = 3 lasting 2.4 d. 

**Figure 7:**
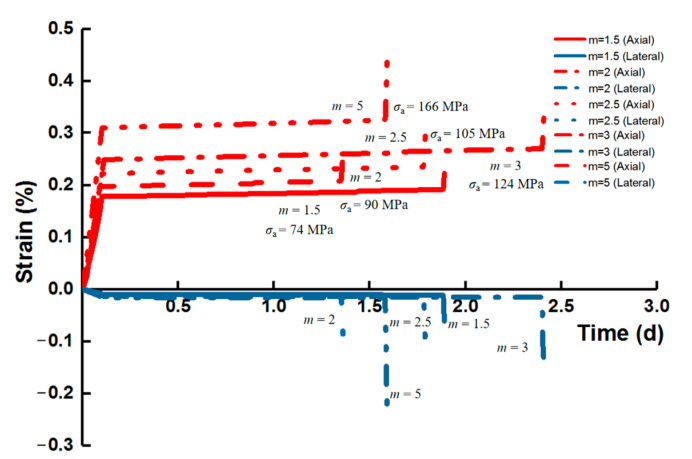
Behavior of creep strain at the last stress level for various homogeneity indexes during uniaxial creep loading.

Under the unconfined creep loading condition, the time-dependent evolution of axial and lateral strains for various *m* is shown in Figure 7. All the specimens exhibited instantaneous elastic deformation after initial loading and then entered into the phase of primary (transient or attenuated) creep, whose duration was extremely short. The strain rate declined rapidly and stayed almost constant at the steady-state (secondary) creep stage. In the end, all the specimens ruptured with the prominent strain accompanied by the increasing strain rate within quite a short period, marking the tertiary (accelerating) creep stage. It can be seen that the transition from the secondary to tertiary creep is not distinct when a sudden failure commenced with dramatic deformation. At the same time, the lateral strain of the specimen was more prominent than the axial strain during the fracturing process.

The last constant applied stress that triggers the creep failure increased with homogeneity index *m*, suggesting that a higher failure strength of rock is enhanced by a higher homogeneity of structural composition. The corresponding axial strain also presented an increasing mode with *m*, which was more notable for the secondary creep, and the lateral deformation displayed the same law as the axial one. 

**Figure 8:**
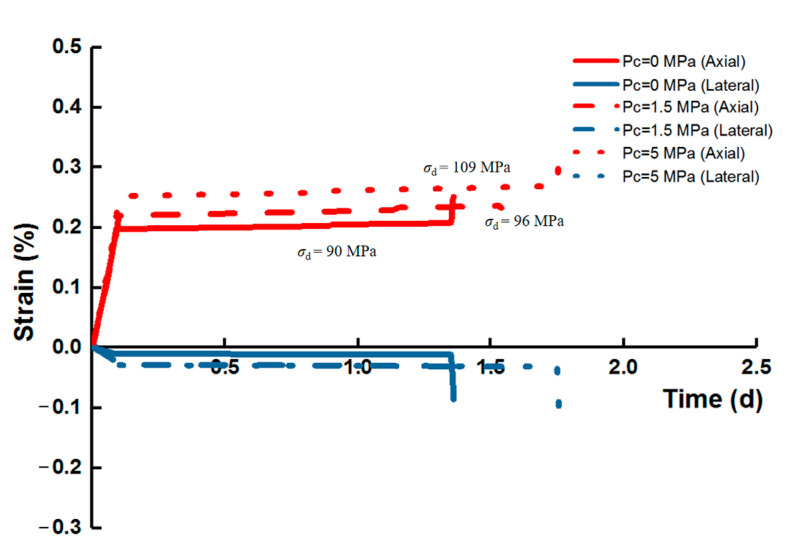
Behavior of creep strain at the last stress level for the constant homogeneity index (*m* = 2).

With the condition where *m* is equal to 2, the variation for axial and lateral creep strains with time under the effect of confining pressure showed a similar tendency to the unconfined case (Figure 8). When the confining pressure increased, both the axial and lateral deformation increased with the deviatoric stress level, and the continuous duration for the last deviatoric stress stage was the longest (*P*_c_ = 5 MPa). 

## 4. Discussions

### 4.1. Evolution Laws of Stress Level vs. Strain Rate

The strain rate during the secondary creep phase is a critical index for the evaluation of long-term stability and creep amount of rock mass. The relationship between applied load level and steady creep rate can be quantitatively represented by:

(9) $$\dot{\varepsilon }=A\sigma_{n}$$

where *A* and *n* are constants, *n* = 3–8 for dislocation creep and *n* is approximately equivalent to 1 for diffusion creep. The parameter *n* is the slope of linear fitting curve (the Log*σ*–Log$\dot{\varepsilon }$ plot) converted from the original expression and is also used for the creep of rocks under compression, sometimes being called the stress corrosion index. 

**Figure 9:**
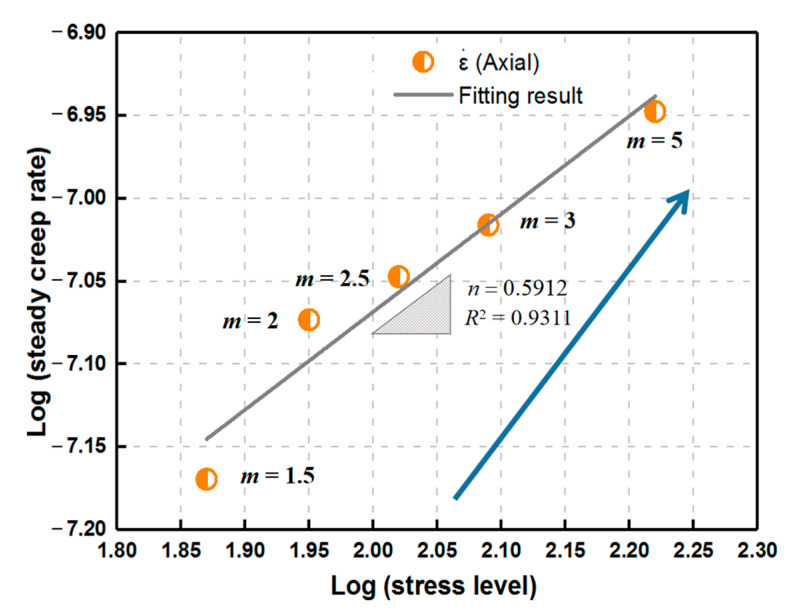
Relationship between the last stress level and steady creep rate for various homogeneity indexes during uniaxial creep loading.

**Figure 10:**
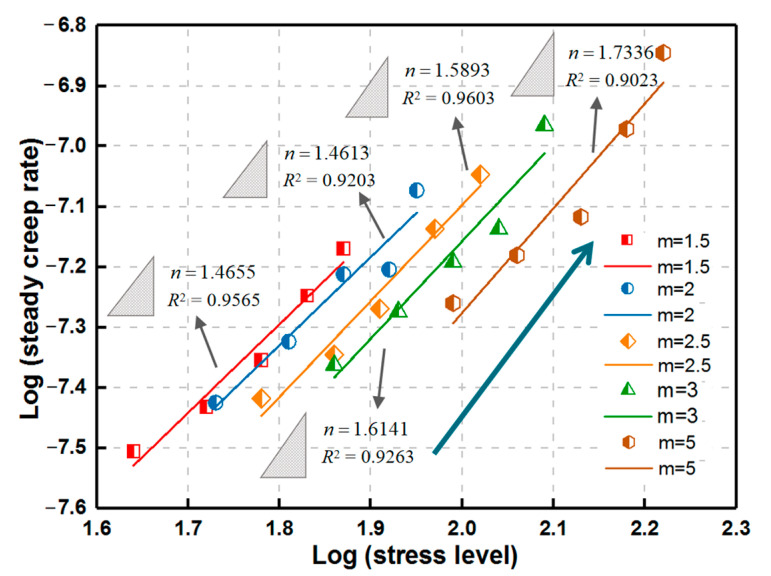
Relationship between stress level and steady creep rate for various homogeneity indexes during uniaxial creep loading.

As presented in Figure 9 and Figure 10, the obtained magnitude order of strain rate reaches 10^−8^/s. All the correlation coefficients *R*^2^ under the unconfined condition are greater than 0.8, which reflects that the corresponding relationships can be well depicted by the model. The fitting relation between the last stress level and axial steady creep rate shows an increasing trend with homogeneity index *m*, indicating that the deformation is more significant for more homogeneous rock material, which further impacts the formation of a damage zone at the accelerating creep stage. For all the specimens, *n* ranges from 1 to 2 and the maximum is 1.7336 for *m* = 5, suggesting that diffusion creep may occur at a low stress level and gradually transition to dislocation creep with the increase in failure strength that is caused by a higher homogeneity of rock. Moreover, for the constant homogeneity index, the steady creep rate increases with stress level and reaches the maximum at the last fixed load that induces failure, which shows that the deformation of rock during the steady-state creep phase depends heavily on the applied stress level.

**Figure 11:**
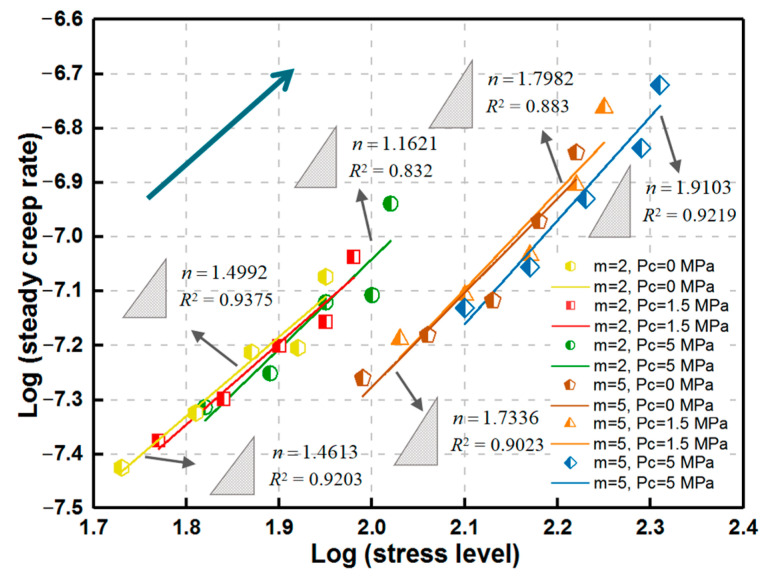
Relationship between stress level and steady creep rate for various homogeneity indexes during triaxial creep loading.

The relationship between deviatoric stress level and axial steady creep rate exhibits a similar increasing law with *m* to the uniaxial case under the same confining condition. *n* increases with *P*_c_ for the same *m* (Figure 11), which indicates that the dislocation creep of rock may be more inclined to occur with the increasing confining pressure. 

**Figure 12:**
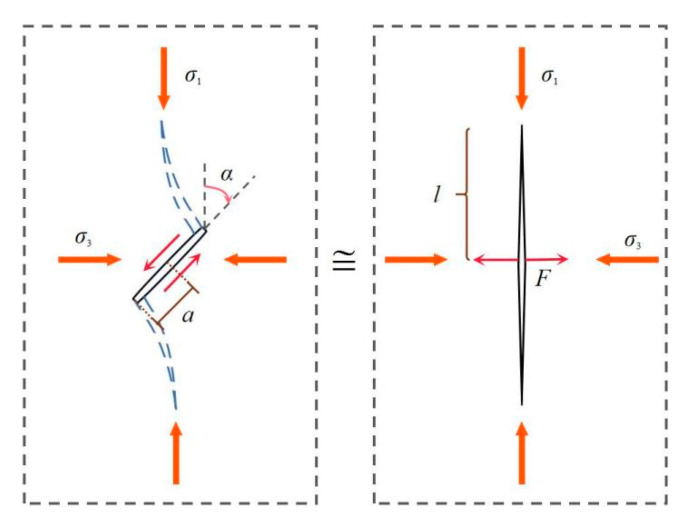
Approximation of an inclined crack with wing cracks by a straight crack with a concentrated force at the center.

The stress level basically moves right by the confining pressure for the invariable homogeneity index, apparently indicating the increase in rock strength under the confining condition. This phenomenon can be further explained by the following model [36], where an inclined crack of the length 2*a*_0_ with wing cracks can be approximated by a straight crack of the length 2*l* with a concentrated force *F* at the center (Figure 12). The stress intensity factor at the crack tips can be described as follows:

(10) $$K_{I}=\frac{F}{\sqrt{\pi l}}-\sigma_{3}\sqrt{\pi l}$$

where

(11) $$F=2a_{0}\left(\sigma_{1}-\sigma_{3}\right)\sin2\alpha \cos\alpha$$

For simplicity, it is assumed that there is no friction between the inclined crack surfaces. Equations (10) and (11) suggest that the stress intensity factor at the wing crack tip decreased by the confining pressure *P*_c_ (*σ*_3_) for the same axial-applied stress *σ*_1_.

### 4.2. Characteristics of Dilatancy

The evolution law of dilatancy is an effective indicator of the instability and failure of brittle rocks. Time-dependent deformation comprises the elastic and inelastic stages where the dilatancy can be reflected by the inelastic volumetric deformation resulting from the development of microcracks in the interior of the rock under different load levels. It is assumed that both elastic modulus *E* and Poisson’s ratio *ν* are the same in all specimen models; the inelastic shear strain $\varepsilon_{q}^{\mathrm{ie}}$ and the inelastic volumetric strain $\varepsilon_{v}^{\mathrm{ie}}$ can be separately expressed as follows:

(12) $$\varepsilon_{q}^{\mathrm{ie}}=\varepsilon_{q}-\frac{2\sqrt{2}}{3}\frac{1+\upsilon }{E}\left(\sigma_{1}-\sigma_{3}\right)$$

(13) $$\varepsilon_{v}^{\mathrm{ie}}=\varepsilon_{v}-\frac{1-2\upsilon }{E}\left(\sigma_{1}+2\sigma_{3}\right)$$

(14) $$\varepsilon_{q}=\frac{2\sqrt{2}}{3}\left(\varepsilon_{1}+\varepsilon_{3}\right)$$

(15) $$\varepsilon_{v}=\varepsilon_{1}+2\varepsilon_{3}$$

and for the uniaxial case (*σ*_3_ = 0 MPa):

(16) $$\varepsilon_{q}^{\mathrm{ie}}=\varepsilon_{q}-\frac{2\sqrt{2}}{3}\frac{1+\upsilon }{E}\sigma_{1}$$

(17) $$\varepsilon_{v}^{\mathrm{ie}}=\varepsilon_{v}-\frac{1-2\upsilon }{E}\sigma_{1}$$

where *ε*_q_ and *ε*_v_ represent the final shear strain and final volumetric strain, respectively.

The relationship between inelastic shear strain and inelastic volumetric strain can be further illustrated by the dilatancy index which is the slope of strain path defining the flow law of rocks during the creep process [11,35]:

(18) $$\frac{1}{DI}=\left|\frac{d\varepsilon_{q}^{\mathrm{ie}}}{d\varepsilon_{v}^{\mathrm{ie}}}\right|$$

where d$\varepsilon_{q}^{\mathrm{ie}}$ and d$\varepsilon_{v}^{\mathrm{ie}}$ are the increment of inelastic shear strain and inelastic volumetric strain, and it should be noted that the former is always positive and, due to the expansion of rock, the latter is always negative. Thus, the absolute value of 1/*DI* is more concise for interpretation.

**Figure 13:**
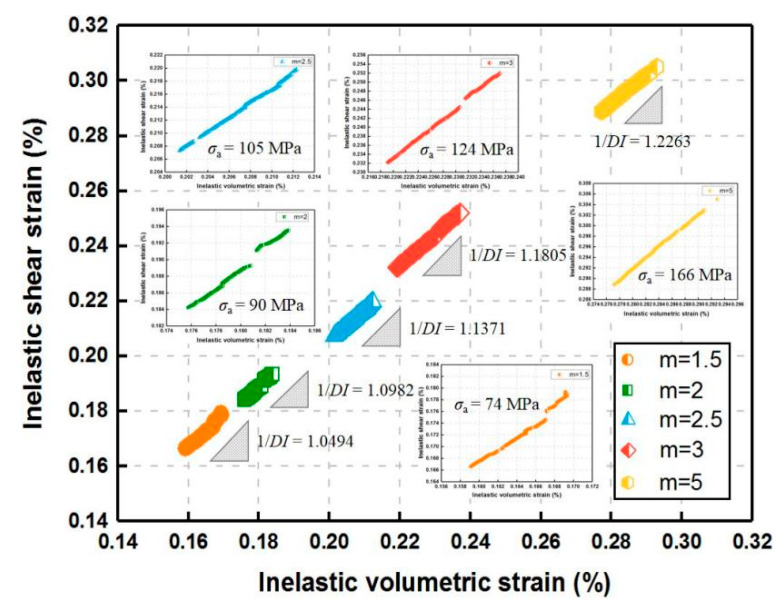
Strain path in inelastic shear strain vs. volumetric strain space for various homogeneity indexes during uniaxial creep loading.

At the last fixed stress, the relationship between inelastic shear strain and inelastic volumetric strain with various m is presented in Figure 13. The strain path displays an almost linear form, suggesting that both the slipping along inclined cracks and the propagation of axial cracks may commence from the initiation of rupture. This is similar to the previously observed experimental phenomena [11,35].

**Figure 14:**
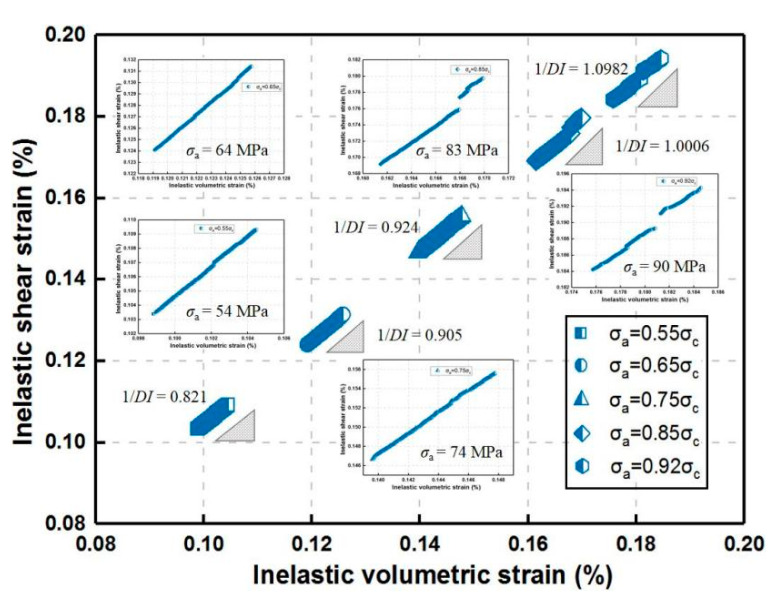
Strain path in inelastic shear strain vs. volumetric strain space for the constant homogeneity index (*m* = 2) during uniaxial creep loading.

As shown in Figure 13, the dilatancy index 1/*DI* increases with the homogeneity index *m*. The reason for this phenomenon is that larger increments of applied stress with higher *m* lead to larger increments of inelastic shear strain, and *DI* declines. For the constant homogeneity index (*m* = 2) (Figure 14), a similar slight decreasing tendency of *DI* is also attributed to the increase in inelastic shear strain increments when the increment-of-stress level ranges from a low to high degree, which indicates that when the rock material approaches failure, *DI* reaches the minimum at a certain high stress level.

**Figure 15:**
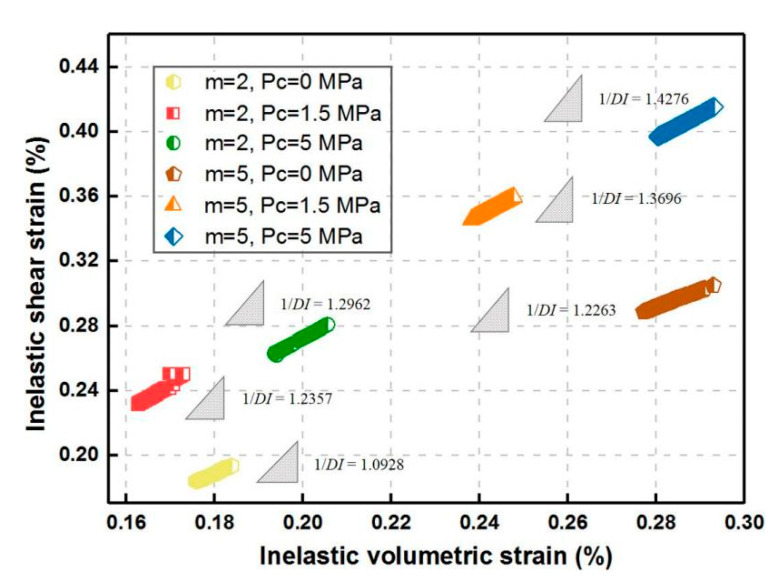
Strain path in inelastic shear strain vs. volumetric strain space for various homogeneity indexes during triaxial creep loading.

Figure 15 presents the variation of 1/*DI* with *m* under the impact of confining pressure. For the same *m*, 1/*DI* gradually increases with *P*_c_. The inelastic volumetric strain in the uniaxial case is smaller than that with *P*_c_ = 5 MPa and larger than that with *P*_c_ = 1.5 MPa, which may result from the close applied load level caused by quite a low confined condition for hard and brittle rock specimen. Furthermore, the evolution law of 1/*DI* with increasing *m* under unchanging confining pressure is similar to the uniaxial case and is more marked than that under the unconfined condition.

### 4.3. Failure Pattern

**Figure 16:**
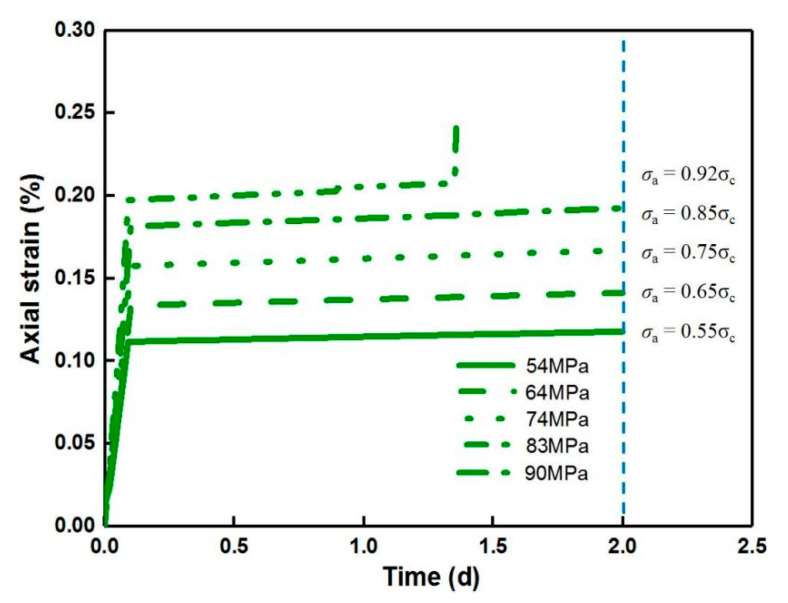
Behavior of creep strain with various stress levels for the constant homogeneity index (*m* = 2) during uniaxial creep loading.

When the homogeneity index *m* is equal to 2, the evolution of creep strain at different stress levels according to a similar increment of 10% *σ*_c_ is displayed in Figure 16. The accelerating creep is not observed until the last stress level surpasses 90% *σ*_c_ (*σ*_a_ = 0.92*σ*_c_), resulting in the ultimate rupture of the rock specimen, which basically agrees with the experimental result by Ma, 2004 [37].

**Figure 17:**
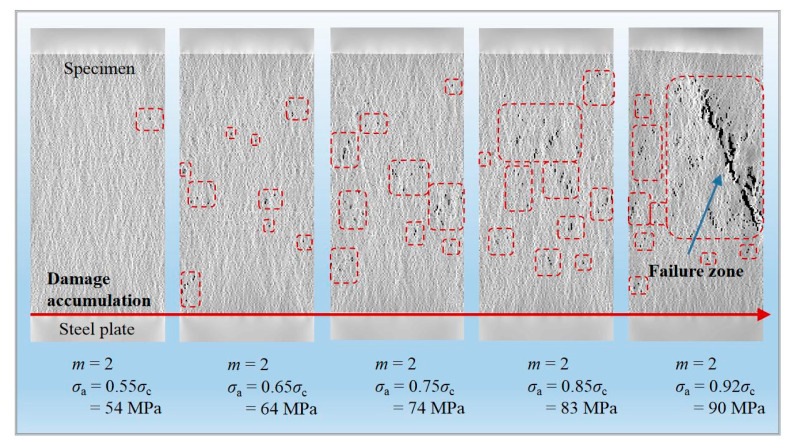
Damage accumulation for the constant homogeneity index (*m* = 2) during uniaxial creep loading.

With the generation of new cracks and the growth of original cracks inside rock specimens, internal damage continuously accumulates, which dominates progressive deformation, leading to a sudden failure. Corresponding to Figure 16, Figure 17 exhibits the damage accumulation process in the interior of rock material with an increasing applied load for *m* = 2. When *σ*_a_ reaches 0.55*σ*_c_ (54 MPa), a few new cracks appear and almost no new damage is generated, showing that the compaction on the microcracks and the specimen stays in a stable state after the self-adjustment of internal stress. At 74 MPa (0.75*σ*_c_), the microcracks tend to aggregate and some local damage zones appear, which indicates that the specimen is starting to enter the unsteady stage. With the further accumulation of damage until *σ*_a_ = 0.92*σ*_c_ (90 MPa), the intensity of microcracks exceeds a critical density, triggering the accelerating creep, during which the microcracks extensively propagate and swiftly coalesce until the macroscopic failure region emerges. It can be found that at low loading stages, the local failure area first appears after reaching the yield strength of the specimen owing to the low homogeneity of internal structure, inducing internal damage accumulation, which further results in the increase in anisotropy of the specimen, and the main fracture zone gradually appears and extends with elapsed time. At the same time, as the applied stress increases, the tensional stress along the axial direction after stress redistribution reaches the tensile strength, producing local axial splitting failure areas.

**Figure 18:**
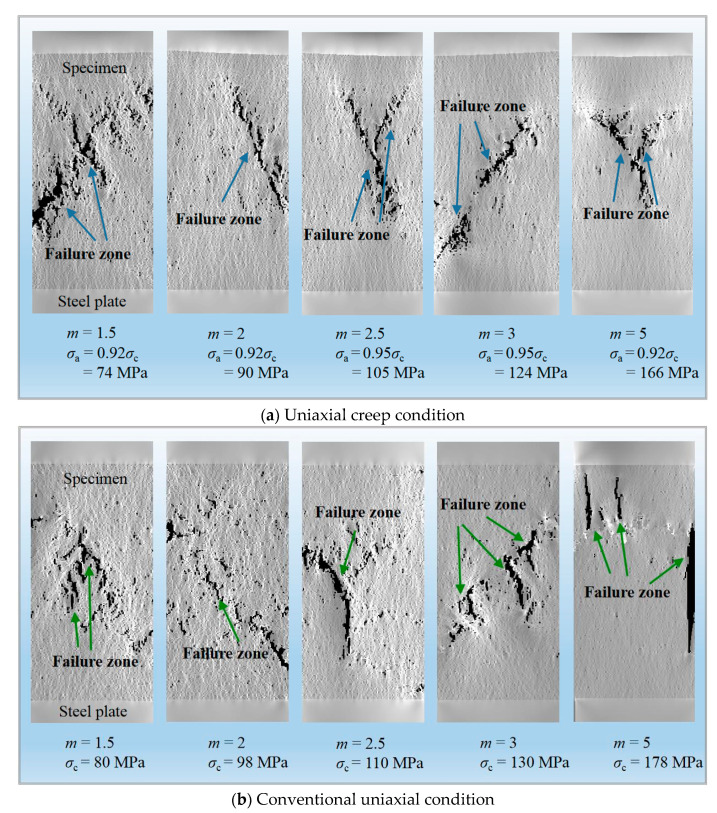
Damage accumulation for various homogeneity indexes under uniaxial creep (**a**) and conventional compression conditions (**b**).

The creep failure pattern for various homogeneity indexes under the unconfined conditions is shown in Figure 18a. Most rock specimens present the nearly penetrated shear-type planes such as the case that *m* = 2, 3, and for *m* = 1.5, 3 and 5, the dominated shear mode is accompanied by partial splitting. The effect of homogeneity on failure pattern after uniaxial creep loading is not conspicuous in this simulation due to the difference in the internal heterogeneity of specimens on the microscopic scale. However, based on the statistical homogeneity from the macroscopic angle, the specimen under conventional compression shows the splitting-based mode compared to the creep condition (Figure 18b), and both cases are similar to the failure pattern of marble described by Zhao et al., 2012 [5], which suggests that the creep failure of rock is a progressive process during which the circulation is between internal damage accumulation and the expansion of weak yield areas, with the continuous evolution of microcracks until eventual rupture. 

**Figure 19:**
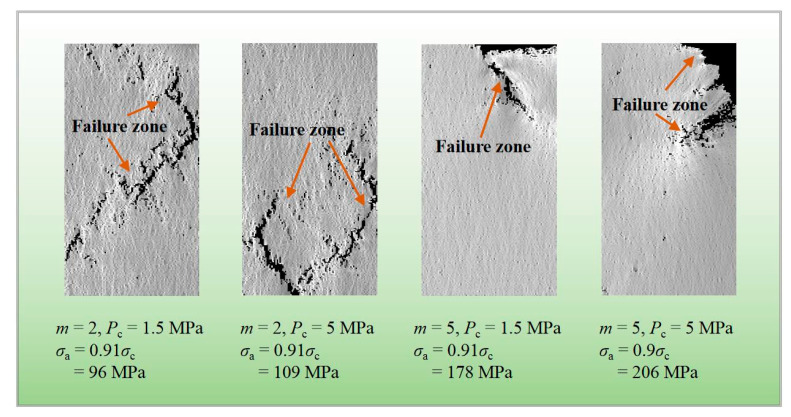
Damage accumulation at the last stress level for various homogeneity indexes during triaxial creep loading.

Under the impact of confining pressure, the creep failure type with *m* = 2 and 5 is shown in Figure 19. Both specimens under the confining pressure of 1.5 and 5 MPa present several macroscopic shear failure regions when *m* = 2. For the higher homogeneity index that is 5, the shear-based form appears in the case where *P*_c_ = 1.5 and 5 MPa, and the rupture is mainly located in the upper region of the specimen, connected with the local heterogeneity of rock under the time effect to some extent. The fracture of the specimen is prone to shear pattern under the confined condition, which is more evident for a higher homogeneity of rock material.

## 5. Concluding Remarks

In order to clarify the effect of homogeneity on the creep properties of brittle rock, the numerical simulation approach, RFPA-Creep (2D), is applied to the comparative analysis on the corresponding time-dependent behavior and failure mode for homogeneity indexes *m* = 1.5, 2, 2.5, 3 and 5. The main conclusions of this study are as follows:

1. During uniaxial creep loading, the deformation is more significant for more homogeneous rock material. Diffusion creep may occur at low stress levels and transition to dislocation creep with increasing applied loads. The increasing law for creep strain with the homogeneity index under an unaltered triaxial condition is similar to the uniaxial case and the dislocation creep may be more inclined to emerge with increasing confining pressure for rock with the same homogeneity.
2. The dilatancy index reaches the maximum at a certain high load level when creep failure happens, and the evolution of dilatancy index with homogeneity index under the same confining pressure is similar to the uniaxial case and is more prominent than that under the unconfined condition.
3. Both the uniaxial and triaxial creep failure are based on the ductile damage accumulation inside rock. The dominant shear-type failure is shown by the uniaxial creep loading manner and the conventional compression case presents the splitting-based failure form. Under the confining pressure, the creep failure mode tends more towards the shear, which is more obvious with a higher homogeneity of rock.

## Figures and Tables

**Table 1:** Rock material parameters of the numerical model.

Homogeneity Index (*m*)	1.5, 2, 2.5, 3, 5 (Uniaxial)
	2, 5 (Triaxial)
Mean compressive strength (*σ*_0_/MPa)	500
Mean elastic modulus (*E*_0_/MPa)	65,000
Poisson’s ratio (*μ*)	0.28
Friction angle (*ψ*/°)	30
Ratio of compression and tension strength (*σ*_c_/*σ*_t_)	10
Coefficient of residual strength	0.1
Attenuation coefficient of strength	0.1
Attenuation coefficient of elastic modulus	0.1
Ratio of long-term strength and short-term strength (*σ*_∞_/*σ*_c_)	0.7

## Data Availability

The data presented in this study are available on request from the corresponding author.
